# Anti-NT5c1A Autoantibodies as Biomarkers in Inclusion Body Myositis

**DOI:** 10.3389/fimmu.2019.00745

**Published:** 2019-04-09

**Authors:** Adam Amlani, May Y. Choi, Mark Tarnopolsky, Lauren Brady, Ann E. Clarke, Ignacio Garcia-De La Torre, Michael Mahler, Heinrike Schmeling, Claire E. Barber, Michelle Jung, Marvin J. Fritzler

**Affiliations:** ^1^Cumming School of Medicine, University of Calgary, Calgary, AB, Canada; ^2^Department of Pediatrics, McMaster University Medical Center, Hamilton, ON, Canada; ^3^Hospital General de Occidente and University of Guadalajara, Guadalajara, Mexico; ^4^PANLAR Myositis Study Group, Guadalajara, Mexico; ^5^Inova Diagnostics, San Diego, CA, United States

**Keywords:** inclusion body myositis, anti-NT5c1A, Mup44, cytosolic 5-nucleotidase 1A, autoantibodies

## Abstract

**Objective:** Sporadic Inclusion Body Myositis (sIBM) is an inflammatory myopathy (IIM) without a specific diagnostic biomarker until autoantibodies to the cytosolic 5′-nucleotidase 1A (**NT5c1A**/Mup44) were reported. The objectives of our study were to determine the sensitivity and specificity of anti-NT5c1A for sIBM, demonstrate demographic, clinical and serological predictors for anti-NT5c1A positivity and determine if anti-nuclear antibody (ANA) indirect immunofluorescence (IIF) staining on HEp-2 cells is a reliable screening method for anti-NT5c1A.

**Methods:** Sera from sIBM patients and controls were stored at −80°C until required for analysis. IgG antibodies to NT5c1A were detected by an addressable laser bead immunoassay (ALBIA) using a full-length human recombinant protein. Autoantibodies to other autoimmune myopathy antigens (Jo-1, OJ, TIF1y, PL-12, SAE, EJ, MDA5, PL7, SRP, NXP2, MI-2) were detected by line immunoassay (LIA), chemiluminescence immunoassay (CIA) or enzyme linked immunosorbent assay (ELISA) and ANA detected by IIF on HEp-2 substrate. Demographic, clinical and serological data were obtained by chart review.

**Results:** Forty-three patients with sIBM, 537 disease control patients with other autoimmune, degenerative and neuromuscular diseases, and 78 healthy controls were included. 48.8% (21/43) of sIBM patients were positive for anti-NT5c1A. The overall sensitivity, specificity, positive predictive value, and negative predictive value of anti-NT5c1A for sIBM were 0.49, 0.92, 0.29, and 0.96, respectively. Compared to sIBM, the frequency of anti-NT5c1A was lower in both the disease control group (8.8%, OR 0.10 [95%CI: 0.05–0.20], *p* < 0.0001) and in the apparently healthy control group (5.1%, OR 0.06 [95%CI: 0.02–0.18], *p* < 0.0001). In the univariable analysis, sIBM patients with more severe muscle weakness were more likely to be anti-NT5c1A positive (OR 4.10 [95% CI: 1.17, 14.33], *p* = 0.027), although this was not statistically significant (adjusted OR 4.30 [95% CI: 0.89, 20.76], *p* = 0.069) in the multivariable analysis. The ANA of sIBM sera did not demonstrate a consistent IIF pattern associated with anti-NT5c1A.

**Conclusions:** Anti-NT5c1A has moderate sensitivity and high specificity for sIBM using ALBIA. The presence of anti-NT5c1A antibodies may be associated with muscle weakness. Anti-NT5c1A antibodies were not associated with a specific IIF staining pattern, hence screening using HEp-2 substrate is unlikely to be a useful predictor for presence of these autoantibodies.

## Introduction

Sporadic Inclusion Body Myositis (sIBM) is one subset of idiopathic inflammatory myopathies (IIM) that is characterized by a clinical presentation of asymmetrical muscle involvement, predominantly affecting the long finger flexors, quadriceps muscles and posterior oropharynx (1, 2). The prevalence of sIBM is thought to range from 4.9 to 33 per million and up to 51.3 per million in those above the age of 50 (1). However, in the absence of a reliable diagnostic biomarker, the prevalence is suspected to be underestimated due to diagnostic challenges in differentiating sIBM from other IIMs (3). Previous reports have suggested diagnostic criteria for sIBM, although the highest diagnostic sensitivity and specificity requires a combination of clinical, electrodiagnostic and pathological assessment (1, 2). Treatment of sIBM is notoriously challenging given the lack of response to conventional immunosuppression (1, 2).

Although sIBM is not responsive to immunosuppression, the immune system is thought to play a significant role in the pathogenesis given documented clonal expansion of T and B cells, findings of plasma cells in pathological specimens, and the association of sIBM with HLA-DR3 (2, 4). Despite this, there are suggestions that sIBM is primarily a myodegenerative disorder involving protein accumulation, vacuolization and various mitochondrial changes (1). The precise pathogenesis of sIBM remains unclear with respect to the interplay of these factors.

**Figure 1:**
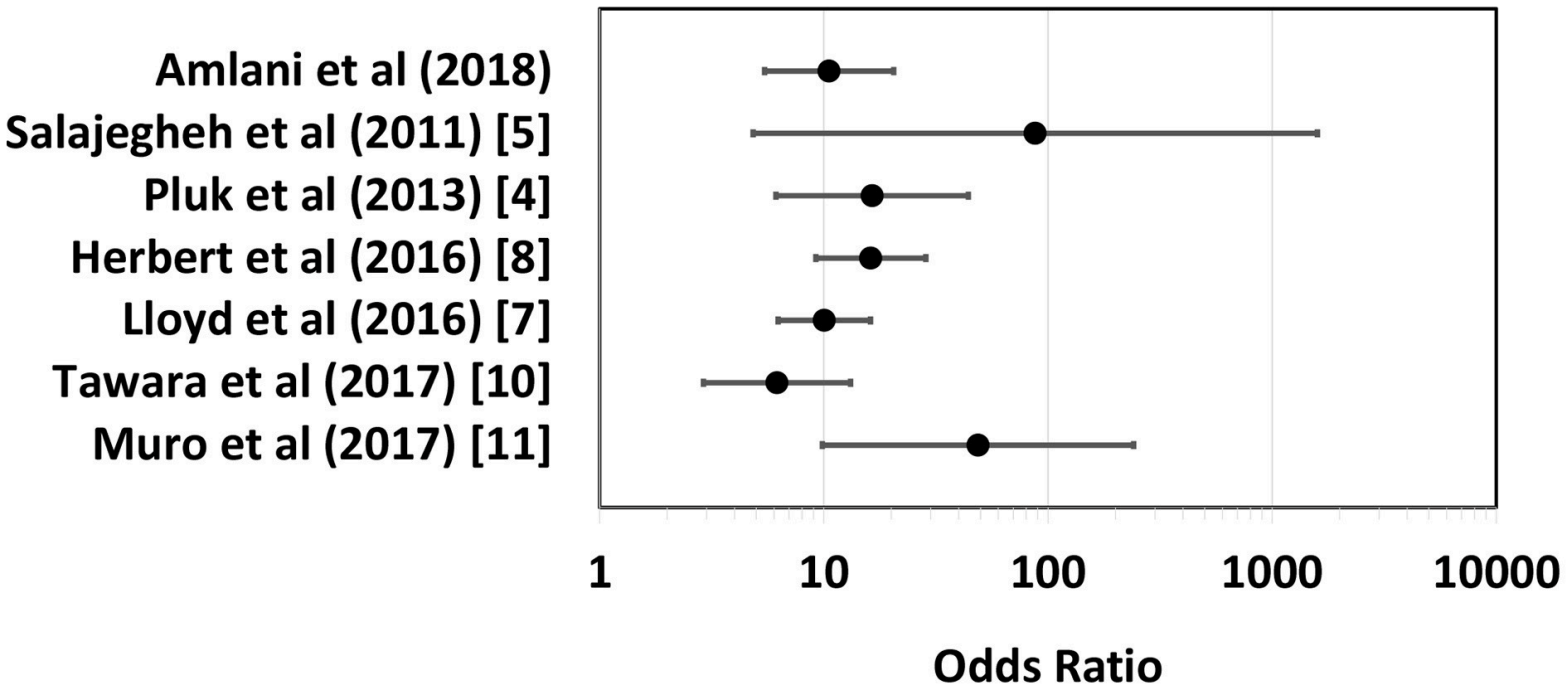
Logarithmic forest plot for diagnostic odds ratios showing data from six anti-NTc51A studies with available sensitivity and specificity data. Error bars indicate 95% confidence intervals. For detailed plot data see Supplemental Table 1.

Unlike other IIMs, up until recently, a biomarker with high sensitivity and specificity for sIBM had yet to be established. In 2011, Salajegheh et al. described a serum autoantibody detected by immunoblot of skeletal muscle lysates that targeted an ~44-kiloDalton (kDa) human **Mu**scle **p**rotein (Mup44) in 52% of sIBM serum samples with 100% specificity (5). In 2013, Larman et al. (6) and Pluk et al. (4) independently identified Mup44, also known as cytosolic 5′-nucleotidase 1A (**NT5c1A**), as the primary autoantibody target. The clinical utility of anti-NT5c1A from a diagnostic, prognostic and treatment perspective in sIBM as previously reported is summarized in Table 1 and odds ratios (OR) with 95% confidence intervals (CIs) of six eligible studies (see section Methods and Supplemental Table 1) are displayed as a logarithmic forest plot in Figure 1. A wide range of sensitivities of anti-NT5c1A antibodies is reported in sIBM ranging from 33 to 80% and it has been suggested that anti-NT5c1A may not be as reliable a biomarker for sIBM as previously thought (8–12, 14). It is also unclear as to whether anti-NT5c1A antibodies are associated with a particular sIBM phenotype, such as increased disease severity, progression, mortality, or response to treatment (2, 8–10). Studies by Larman et al. indicated that anti-NT5c1A was associated with a 6- to 9-fold increase in the likelihood of having sIBM as compared to polymyositis and proposed possible advantages of avoiding unnecessary steroid treatment and/or invasive muscle biopsies (6). Mastaglia and Needham included anti-NT5c1A in a proposed diagnostic algorithm for sIBM (15); however, this biomarker appeared to only provide a supportive diagnostic role as opposed to pointing to pathognomonic features of sIBM, again highlighting the necessity of a muscle biopsy.

**Table 1:** Overview of results compared to results of published studies on frequency of anti-NT5c1A in various diseases and controls.

**References**	**Immunoassay**	**IBM** **(*n*)**	**PM/DM** **(*n*)**	**SLE** **(*n*)**	**SjS** **(*n*)**	**SSc** **(*n*)**	**OA** **(*n*)**	**RA** **(*n*)**	**MCTD** **(*n*)**	**NMD** **(*n*)**	**JM** **(*n*)**	**JIA** **(*n*)**	**DC** **(*n*)**	**HC** **(*n*)**	**Comments**
Amlani et al. (this study)	ALBIA with full length human recombinant protein	48.8% (43)	7% (142)	13.6% (199)	0% (19)	6% (50)	10.6% (47)	0% (27)		15.4% (13)	JDM 0% (40)		8.8% (537)	5.1% (78)	Current study
Salajegheh et al. (5)	IB muscle lysate	52% (25)	0% (25)										0% (25)	0% (15)	
Pluk et al. (4)	IP of *in vitro* TnT protein	33% (94)	PM 4.5% (22) DM 4.2% (24)							3.2% (94)			3.6% (140)	0% (32)	Three non-contiguous epitopes: aa221-243 primary epitope
Goyal et al. (7)	WB screen then confirmed by ELISA	72% (25)													More severe motor, bulbar and respiratory involvement
Herbert et al. (8)	ELISA 3 peptides representing major epitopes (4)	37% (238)	4.3% (185)	20.5% (44)	36.4% (22)	2.3% (44)		2.3% (44)		4.3% (93)			3.5% (458)		
Lloyd et al. (9)	WB of lysates from transfected HEK293 cells	60.6% (117)	PM 4.8% (42) DM 15.1% (159)	13.5% (96)	22.7% (44)								14.4% (341)	4.8% (42)	Muscle Pathology: Lower prevalence of rimmed vacuoles in antibody positive patients.
Tawara et al. (10)	CBA transfected COS cells	35.8% (67)	PM 13.9% (36) DM 12.9% (31)	0% (nr)	0% (nr)					0% (16)			8.8% (147)	0% (10)	Frequency of hepatitis C virus antibodies lower; mean area of type 2 myofibers was smaller
Lilleker et al. (11)	ELISA 3 peptides representing major epitopes (4)	32.8% (311)													higher adjusted mortality risk, lower frequency of proximal upper limb weakness at disease onset, increased prevalence of excess of cytochrome oxidase deficient fibers on muscle biopsy analysis
Muro et al. (12)	ELISA with IP	80% (10)	PM 10% (10) DM 11.1% (144)	6% (50)	4% (50)	8% (50)			0% (10)		JDM 16.7% (12)		8.3% (314)	2.4% (42)	
Yeker et al. (13)	IB of HEK cell lysates										26.8% (380)	26.7% (30)		12% (92)	Anti-NT5C1A autoantibody-positive myositis had greater pulmonary symptoms at diagnosis, more frequent hospitalizations and required a larger number of medications
Rietveld et al. (14)	ELISA			10.3% (252)	11.9% (193)										

*ALBIA, addressable laser bead immunoassay; CBA, cell based assay; COS, CV-1 in Origin with SV40 genetic material; DC, disease controls; ELISA, enzyme linked immunosorbent assay; HC, healthy controls; HEK, human embryonic kidney; IB, immunoblot; IIM, idiopathic inflammatory myopathy; IBM, inclusion body myositis; IP, immunoprecipitation; JIA, juvenile idiopathic arthritis; JM, juvenile myositis; MCTD, mixed connective tissue disease; n, number of subjects; nr, not reported; NMD, neuromuscular diseases; nr, not reported; OA, Osteoarthritis; RA, Rheumatoid Arthritis; SjS, Sjögren's syndrome; SLE, systemic lupus erythematosus; SSc, systemic sclerosis; TnT, transcription and translation of in vitro synthesized protein; WB, western immunoblot*.

The primary aim of our study was to investigate the sensitivity and specificity of anti-NT5c1A for sIBM using an addressable laser bead immunoassay (ALBIA) and a purified, full-length human recombinant protein. The use of an ALBIA as a diagnostic assay platform has advantages over other platforms, such as ELISA and immunoblot in that has potential to be multiplexed (16, 17) and hence akin to other multiplexed technologies (18) the capability of being used as a diagnostic array containing the main target autoantigens associated with IIM. Patients without sIBM including apparently healthy individuals and those with other neuromuscular and systemic autoimmune rheumatic diseases (SARDs) served as controls. We also set out to determine if these autoantibodies are associated with a specific indirect immunofluorescence (IIF) staining pattern on HEp-2 substrates as well as clinical and demographic factors.

## Methods

### Patients and Controls

Patients with a diagnosis of sIBM, IIM and disease controls as identified below were followed by tertiary care specialists at McMaster University (Hamilton, Ontario Canada), the University of Calgary (Calgary, AB, Canada) or the University of Guadalajara (Mexico). The diagnosis of IIM (19, 20), juvenile dermatomyostis (JDM) (21), and sIBM (22) was made in accord with published criteria. Patients fulfilling the American College of Rheumatology (ACR) or Systemic Lupus International Collaborating Clinics (SLICC) Classification Criteria for systemic lupus erythematosus (SLE) (23, 24) were part of the Southern Alberta Registry for Lupus Erythematosus (STARLET) and another local SLE cohort (Calgary, Canada). Diagnosis of other disease comparator controls was based on specialist co-author's judgment. Sera from healthy controls were from a biobank of blood donors matched for the age range and sex of the sIBM cohort. Osteoarthritis (OA) sera were from a biobank used in a previous study (25). Sera were collected and biobanked at Mitogen Advanced Diagnostics Laboratory (Calgary, AB, Canada) at −80°C until required for analysis. The research was approved by the Health Research Ethics Boards (HREB) at each institution, conducted in accordance with the Helsinki Declaration and where required by the HREB written informed consent was obtained.

Sera of 580 patients with various comparator diseases and 78 healthy control patients were analyzed for anti-NT5c1A positivity. Clinical diagnoses (Table 2) included sIBM (*n* = 43), IIM (*n* = 142), SLE (*n* = 199), systemic sclerosis (SSc) (*n* = 50), osteoarthritis (*n* = 47), congenital or acquired neuromuscular or metabolic disorders (*n* = 13), rheumatoid arthritis (*n* = 27), Sjögren's syndrome (SjS) (*n* = 19), and JDM (*n* = 40).

**Table 2:** The frequency and odds ratio of anti-NT5c1A antibodies in controls and other systemic autoimmune rheumatic diseases compared to sporadic inclusion body myositis.

**Disease**	**N**	**Frequency of anti-NT5c1A (%)**	**Odds ratio**	**95% confidence interval**	*p*-Value^*^
sIBM	43	21 (48.8)	–	–	–
IIM	142	10 (7.0)	0.08	0.03, 0.19	<0.0001
SLE	199	27 (13.6)	0.16	0.08, 0.34	<0.0001
SSc	50	3 (6.0)	0.07	0.02, 0.25	<0.0001
OA	47	5 (10.6)	0.12	0.04, 0.38	0.0001
NMD	13	2 (15.4)	0.19	0.04, 0.96	0.03
RA	27	0 (0.0)	–	–	<0.0001
SjS	19	0 (0.0)	–	–	0.0002
JDM	40	0 (0.0)	–	–	<0.0001
HC	78	4 (5.1)	0.06	0.02, 0.18	<0.0001

* *p-Value for two-sample test of proportions compared against sIBM, p < 0.05 considered to be statistically significant. HC, healthy controls; IIM, inflammatory immune myopathies; JDM, juvenile dermatomyositis; NMD, neuromuscular/metabolic disorders; OA, osteoarthritis; RA, rheumatoid arthritis; sIBM, sporadic inclusion body myositis; SjS, Sjögren's syndrome; SLE, systemic lupus erythematosus; SSc, systemic sclerosis*.

### Autoantibody Testing

#### Coupling of Addressable Laser Beads

Autoantibody testing was performed at Mitogen Advanced Diagnostics Laboratory. Antibodies to NT5c1A were detected by ALBIA using a full-length human recombinant protein (Origene, Rockville, MD: Cat. #TP324617) and protocols adapted from previous studies (26, 27). Unless otherwise specified, all incubations and reactions were conducted at room temperature. 5.0 × 10^6^ addressable laser beads (Luminex Corp., Austin, TX, USA) were pipetted into micro tubes (USA Scientific Inc., Ocala, FL, USA) and placed into a magnetic separator (DynaMag-2; Life Technologies, Carlsbad, CA, USA) for 1 min followed by removal of the fluid supernatant. 80 μl of activation buffer (100 nM monobasic sodium phosphate, pH 6.2., Thermo Scientific, Waltham, MA, USA) was added and the beads were resuspended by gentle sonication and votexing followed by addition of 10 μl of 50 mg/ml sulfo-NHS (N-hydroxysuccinimide, Thermo Scientific) and 10 μl of 5 mg/ml EDC (1-ethyl-3-(3-dimethylaminopropyl) carboiimide hydrochloride (Thermo Scientific). The beads were then sonicated and vortexed again followed by a 20 min incubation in the dark at room temperature. After incubation, the beads were washed twice with coupling buffer [50 mM 2-(N-Morpholino) ethanesulfonic acid hydrate, pH 5.0], followed by the addition of the optimal amount of recombinant NT5c1A protein (diluted in coupling buffer) as previously determined by checkerboard analysis using various amounts of the recombinant NT5c1A coupled to the beads to provide optimal signals of high sensitivity and specificity as determined by receiver operator characteristic. The coupled beads were incubated at room temperature for 2 h by rotation followed by two washes with PBS-TBN wash/storage buffer (PBS, 0.1% BSA, 0.02% Tween-20, 0.05% azide, pH 7.4). 500 μl of wash/storage buffer was added back to the coupled beads and stored at 4C in the dark until required for use.

#### Anti-NT5c1A Assay

2.0 × 10^4^ of the coupled beads diluted in HRP sample diluent (Inova Diagnostics, San Diego, CA, USA) was pipetted into each well of a 96 well plate (Thermo Scientific., Waltham, MA, USA) followed by the addition of 5 μl of patient sera diluted at 1:100 in HRP sample diluent, and then incubated for 30 min on an orbital shaker. The fluid phase was decanted by placing the plates in a Handheld Magnetic separator block (Millipore Sigma, Burlington MA, USA) for 1 min, followed by the addition of two changes of the wash buffer (Hemosil Rinse, Inova Diagnostics., San Diego, CA, USA). 50 μl of phycoerythrin-conjugated goat anti-human IgG (Jackson ImmunoResearch Laboratories, Inc., West Grove, PA, USA) diluted at 1/200 in sheath fluid (Luminex Corp., Austin, TX, USA) was added to each well and incubated on the orbital shaker for 30 min following by two washes with wash buffer. Control positive and negative samples were included in each run and plates were analyzed with the MagPix® or Luminex 100 luminometers (Luminex Corp., Austin, TX, USA). The data was expressed as median fluorescence units (MFU) and the cut-off for anti-NT5c1A established at 600 MFU, which was two standard deviations above the mean of the healthy controls.

#### Validation

The ALBIA was validated using a commercially available ELISA kit (Euroimmun GmbH, Luebeck, Germany), which was a licensed version of a previously published assay that utilized synthetic NT5c1A peptides as analytes (8, 11). Side by side comparison of the ALBIA to the ELISA showed an overall agreement of positive and negative test results of 93.75% (data not shown).

Autoantibodies associated with IIM (Jo-1, PL-7, PL-12, Mi2, Mi2-α, Mi2β, MDA5, NXP2, TIF1γ, Ku, EJ, OJ, PM/Scl-100/PM/Scl-75, Ro52/TRIM21) were detected by a line immunoassay (LIA: Euroimmun GmbH, Lübeck, Germany) and those associated with necrotizing autoimmune myopathy (Signal Recognition Particle (SRP), 3-hydroxy-3-methyl-glutaryl-coenzyme A reductase (HMGCR) by chemiluminescence immunoassay (CIA) or ELISA (Inova Diagnostics, San Diego, CA). Autoantibodies to the survival of motor neuron and gemin3 (anti-SMN) were tested by ALBIA on a Luminex 200 flow fluorometer (Luminex Corp., Austin TX, USA) as previously described (28). Other autoantibodies directed against U1-RNP, SSA/Ro60, and dsDNA were identified by ALBIA (FIDIS Connective 13, TheraDiag, Paris, France).

Anti-nuclear antibodies (ANA) were detected by IIF on HEp-2 substrates (NOVA Lite HEp-2, Inova Diagnostics) at a dilution of 1/80 and read on an automated instrument (NOVA View, Inova Diagnostics) which interpolates fluorescence intensity to an end point titer (29). IIF staining patterns were classified according to the International Consensus on Autoantibody Patterns (ICAP: www.anapatterns.org) (30). If multiple patterns were noted in individual sera, they were recorded as separate ICAP patterns (https://anapatterns.org/index.php).

### Statistical and Clinical Analysis

The Chi-squared test was used to determine the difference in proportions of anti-NT5c1A positivity in patients with sIBM compared with other diseases and healthy controls. A *p*-value of <0.05 was considered to be statistically significant. The sensitivity, specificity, positive predictive value, negative predictive value, and positive and negative likelihood ratios for anti-NT5c1A for sIBM were calculated and also displayed as a receiver operator characteristic (ROC). Univariable logistic regression was used to assess the relationship between anti-NT5c1A and demographic (sex, age), biochemical (creatine kinase (CK) level) and clinical (presence of dysphagia, objective assessment of severity of weakness) characteristics. Muscle weakness and dysphagia were chosen *a priori* for inclusion in a multivariable logistic regression model for disease severity. The canonical features of the disease are quadriceps and deep finger flexion weakness for which quantitative measurements were recorded, and swallowing studies were performed on all symptomatic patients. As previously published, a BIODEX isokinetic dynamometer was used to quantify knee flexor strength and was graded as <10 Newton meters (Nm) = severe, 10–30 Nm = moderate, 30–60 Nm = mild (31). The relationship between anti-NT5c1A and ANA IIF pattern was also assessed using logistic regression with the following categories: presence of any nuclear pattern, only nuclear patterns, cytoplasmic patterns, mitotic patterns, all negative, cytoplasmic and/or mitotic pattern (CMP) only, and negative CMP. All analyses were conducted using the Statistical Package for the Social Science (SPSS) version 24 software. A literature review performed on PubMed using the key words “inclusion body myositis,” “Mup44,” and “NT5c1A” identified 10 studies but only 6 had assessed sensitivity and specificity of anti-NT5c1A antibodies. Along with data from the present study, data from each of these 6 publications was used to calculate OR with 95% CIs (Supplemental Table 1) and displayed as a logarithmic forest plot (Figure 1).

## Results

**Figure 2:**
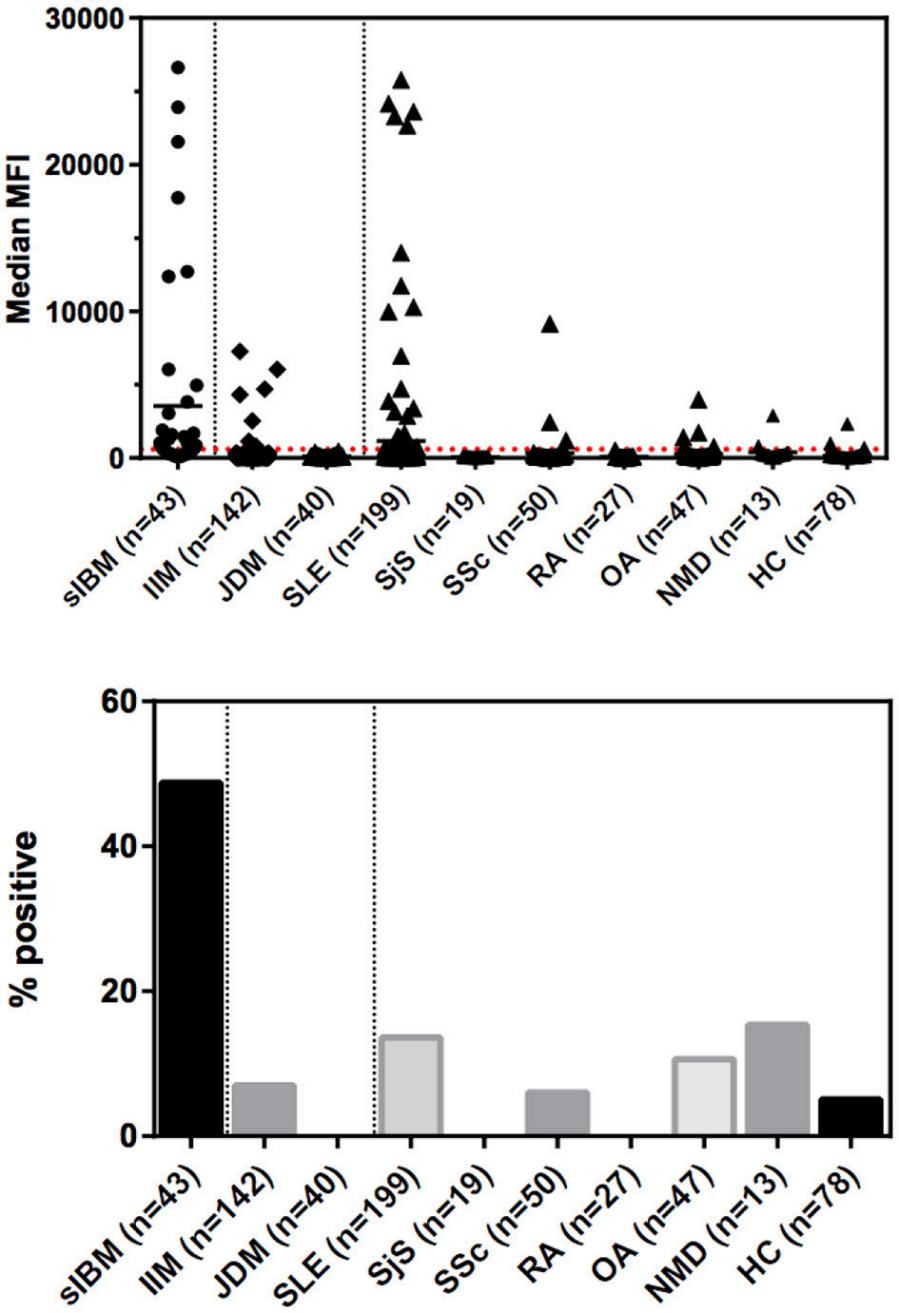
Levels and prevalence of anti-NT5c1a antibodies in sporadic inclusion body myositis (sIBM) and other conditions as detected by an addressable laser bead immunoassay based on full-length human recombinant antigen. The cutoff was established at 600 MFU (2 standard deviations above the mean of healthy controls; see dotted line) which provided a diagnostic sensitivity of 48.8% and a specificity of 91.8%. Horizontal bars indicate the mean values for each group. DM, dermatomyositis; HC, healthy controls; IIM, idiopathic inflammatory myopathies; JDM, juvenile dermatomyositis; MFI, median fluorescence intensity/median fluorescence units; NMD, neurological/metabolic disorders; OA, osteoarthritis; RA, rheumatoid arthritis; sIBM, sporadic inclusion body myositis; SjS, Sjögren's syndrome; SLE, systemic lupus erythematosus; SSc, systemic sclerosis.

**Figure 3:**
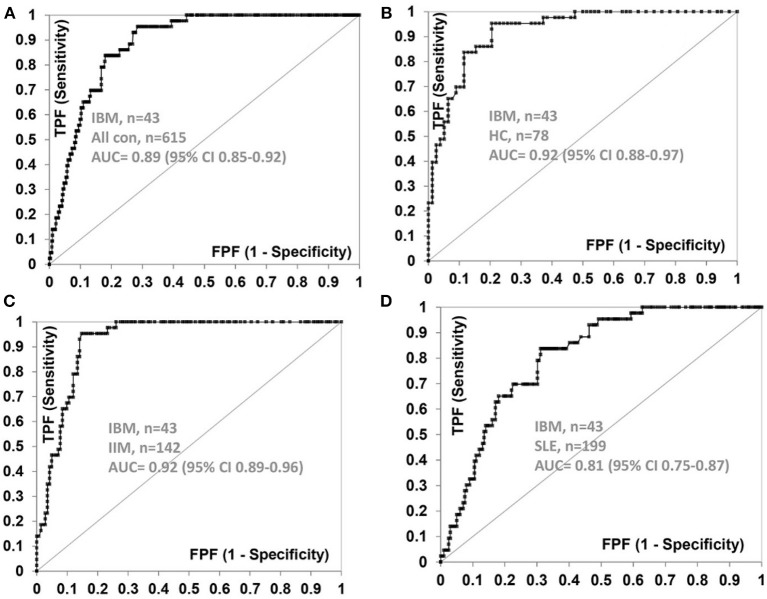
Receiver operator characteristic (ROC) curve for anti-NT5c1A antibodies with sIBM (*n* = 43) as the true state against **(A)** all control sera (*n* = 615), **(B)** healthy controls (*n* = 78), **(C)** idiopathic inflammatory myopathies (*n* = 142), and **(D)** systemic lupus erythematosus (*n* = 199). AUC, area under the curve; CI, confidence interval; FPF, false positive fraction; HC, healthy controls; IIM, idiopathic inflammatory myopathies; SLE, systemic lupus erythematosus; TPF, true positive fraction.

The frequency of anti-NT5c1A in various comparator diseases and healthy controls is summarized in Table 2 and illustrated in Figure 2. Demographics of the sIBM cohort (Table 3) included a median age of 70.0 years (interquartile range (IQR) 60.0, 75.0) with 37.2% females and a median CK level of 444.0 U/L (IQR 252.8, 622.3). The frequency of anti-NT5c1A in the sIBM group was 21/43 or 48.8% [95% CI: 33.9, 63.8]; while the frequency of anti-NT5c1A in the non-sIBM disease comparator groups was 8.8% ([95% CI: 6.4, 11.1]) and in the healthy control group was 5.1% ([95% CI: 0.2, 10.0]). The frequency of anti-NT5c1A in each comparator group was lower than in the sIBM group (*p* < 0.05, Table 2). In fact, there was no positive anti-NT5c1A sera identified within the RA, primary SjS, or JDM cohorts. The overall sensitivity, specificity, positive predictive value, and negative predictive value of anti-NT5c1A in sIBM were 0.49, 0.92, 0.29, and 0.96, respectively. When ROC analysis was used to compare sIBM vs. all controls including healthy controls, healthy controls alone, other IIM, and SLE, the areas under the curve (AUC) were 0.89 [95% CI: 0.85–0.92], 0.92 [95% CI: 0.88–0.97], 0.92 [95% CI: 0.89–0.96] and 0.81 [95% CI: 0.75–0.87], respectively (Figure 3).

**Table 3:** Clinical and demographic features of sIBM patients.

**Gender**	**Age**	**CK**	**Anti-NT5c1A titer** (MFU)^*^	**Dysphagia (Y/N)**	**Severity** **(1 = Mild, 2 = Moderate, 3 = Severe)**
F	54	732	276	Y	2
M	83	812	576	Y	2
F	82	163	1457	Y	3
M	79	170	3815	Y	1
M	79	533	3042	Y	2
F	76	792	400	Y	3
M	75	419	6034	Y	3
M	75	219	869	Y	2
F	73	486	803	Y	2
F	73	250	23911	Y	2
M	71	153	387	Y	1.5
F	71	175	1312	Y	2.5
M	70	490	278	Y	2
F	70	N/A	246	Y	1
F	69	476	1594	Y	1.5
M	67	669	740	Y	2.5
M	65	329	1683	Y	2
F	62	796	336	Y	2
M	60	N/A	592	Y	3
F	57	1265	26623	Y	2
M	56	363	17748	Y	2.5
F	56	710	1903	Y	3
M	54	261	112	Y	1
M	86	355	151	N	2
M	81	110	331	N	2
M	76	703	441	N	1
M	76	279	184	N	1
F	75	232	157	N	1.5
M	74	391	99.5	N	2
F	74	901	12370	N	1
F	73	215	649.5	N	1.5
M	72	305	518	N	1.5
M	72	502	12706	N	2
M	69	469	256	N	2
M	67	624	283	N	2
M	67	500	164	N	1.5
F	67	310	157	N	2
M	60	N/A	477	N	2
M	58	481	4960	N	2
M	55	367	1039	N	2
M	54	596	276	N	1.5
F	54	617	1023	N	3
M	53	204	21563	N	3
37.2% female	Median: 70.0 IQR 60.0,75.0	Median: 444.0 IQR 252.8, 622.3	Median: 592.0 IQR 276.0, 1903.0	53% had dysphagia	Median: 2.0 IQR 1.5, 2.1

* *Shaded cells indicate positive results of >600 MFU (cutoff). F, female; IQR, interquartile range; M, male; MFU, median fluorescence units; NA, not available; N, no; Y, yes*.

Within the sIBM patients, univariable analyses of the association between demographic, serological, and clinical features and anti-NT5c1A demonstrated that patients with higher muscle weakness were more likely to be anti-NT5c1A positive (OR 4.10 [95% CI: 1.17, 14.33], *p* = 0.027) (Supplemental Figure 1), but there was no association with age (OR 0.98 [95% CI: 0.92, 1.04], *p* = 0.508), male sex (OR 0.41 [95% CI: 0.12, 1.47], *p* = 0.172), CK levels (OR 0.97 per 100 U/L [95% CI: 0.76, 1.25], *p* = 0.839) or presence of dysphagia (OR 2.89 [95% CI: 0.83, 10.02], *p* = 0.09). Multivariable analyses found a trend toward higher likelihood of anti-NT5c1A positivity in patients with higher disease severity as judged by muscle weakness (OR 4.30 [95% CI: 0.89, 20.76], *p* = 0.069) but there was no association with age (OR 0.96 [95% CI: 0.88, 1.04], *p* = 0.331), male sex (OR 0.44 [95% CI: 0.91, 2.09], *p* = 0.300), CK levels (OR 0.89 [95% CI: 0.64, 1.22], *p* = 0.64) or presence of dysphagia (OR 2.06 [95% CI: 0.45, 9.49], *p* = 0.448). In the SLE cohort of 199 patients, only 2 (0.1%) had documented myositis and neither of these patient's sera were positive for anti-NT5c1A antibodies.

Forty-three sIBM patients were examined for ANA, including CMP IIF patterns on HEp-2 substrate (Supplemental Table 2). Univariable and multivariable analysis did not demonstrate any association between nuclear, mixed nuclear, cytoplasmic, mitotic, mixed cytoplasmic and mitotic, or negative IIF patterns with NT5c1A positivity in sIBM sera (data not shown). For the anti-NT5c1A positive sIBM sera, 15/21 (71.4%) had a nuclear pattern, 10/21 (47.6%) had a cytoplasmic pattern, 6/21 (28.6%) had a mitotic cell pattern and 2/21 (9.5%) had no nuclear or CMP staining (i.e., ANA negative). Note that total percent above is >100 because some samples had more than one staining pattern. For example, only 6/21 (28.6%) had only a nuclear staining pattern, 4/21 (19%) had only CMP staining, while 9/21 (42.9%) had mixed nuclear and CMP staining. A breakdown of the nuclear staining patterns revealed that the majority had a speckled pattern and of those with cytoplasmic staining, the majority had a discrete speckled pattern. Similarly, analysis of ANA IIF in SLE sera that were anti-NT5c1A positive did not reveal a consistent or distinctive pattern of staining. With regards to other IIM-related antibodies, three (7%) of the sIBM patients had anti-SMN and one had anti-HMGCR antibodies. The sIBM patients had negative autoantibody tests to the remainder of the IIM-related antigens (Jo-1, OJ, TIF1γ, PL-12, SAE, EJ, MDA5, PL7, SRP, NXP2, MI-2) (Supplemental Table 2).

## Discussion

In our study, anti-NT5c1A had a moderate sensitivity and high specificity for sIBM. Previous studies have reported the sensitivity of anti-NT5c1A in sIBM to be 0.33–0.80 (4–6, 8–12) (summarized in Table 1). The variability is likely due to technical aspects (i.e., cutoff thresholds and antigen purity) of the various immunoassays employed, patient selection criteria and other demographic variables. The specificity of anti-NT5c1A for sIBM in the context of IIM and other neuromuscular disorders has been reported in the range of 92–100% (see Table 1). This high specificity was challenged when more recent data showed that anti-NT5c1A was found in other autoimmune conditions, particularly in patients with SLE (4–20%) and SjS (6–36%) (8, 9, 12, 14). Yeker et al. using a western blotting technique with lysates of NT5c1A transfected HEK (human embryonic kidney) cells reported anti-NT5c1A in 27% of JDM (*n* = 380) and juvenile idiopathic arthritis (*n* = 30) patients (13). In our SLE cohort, 27/199 (13.6%) patients were also found to be positive for anti-NT5c1A, 8/199 (4%) had remarkably high titers >10,000 MFU (Figure 2). In contrast, none of our SjS (*n* = 19) or JDM (*n* = 40) patients were anti-NT5c1A IgG positive. Although a systematic review of the SLE patients in our cohort for the presence of muscle weakness has not been done, only 2 had documented clinically apparent myositis and neither of these had anti-NT5c1A antibodies.

Our findings support the suggestion that anti-NT5c1A has diagnostic utility alongside other clinical and pathological evidence of sIBM, especially because symptom profiles and muscle biopsy findings can be inconclusive (1). However, our current observations, along with other reports of a significant frequency of anti-NT5c1A in SjS and SLE, suggest that a positive test will be difficult to interpret in patients who present with non-specific complaints of myalgia, fatigue and/or weakness (2). Although univariate analysis in our study showed that anti-NT5c1A was associated with muscle weakness in our sIBM patients, these findings are not necessarily applicable to SLE or other conditions. Hence, additional studies will be required to determine if there is a unique clinical phenotype associated with anti-NT5c1A in these other conditions that either overlaps with or is distinctive from sIBM.

Screening for anti-NT5c1A antibodies in sIBM patients by ANA IIF will also be challenging. In keeping with many other autoantibodies described in IIM (32), anti-NT5c1A antibodies were not associated with a specific IIF staining pattern on HEp-2 cells at a 1/80 screening serum dilution. It has been suggested that the detection of some IIM-related autoantibodies can be improved by decreasing the serum dilution to 1/40 (33), although the decreased specificity at that dilution may not be an optimal approach either (32). Hence, the use of the IIF test on HEp-2 substrates as a screen for anti-NT5c1A antibodies is unlikely to be a useful serological screening test to detect the presence of these autoantibodies.

The presence of anti-NT5c1A in various SARDs suggests a B-cell response that is a pathophysiological feature held in common with sIBM and perhaps unrelated to the myodegenerative features of sIBM (11). A common feature of these conditions may also be attributed to genetic or epigenetic associations within shared variants of the human leukocyte antigen (HLA) locus (12). In addition, sequencing of DNA from sIBM patients has interestingly identified rare missense variants in proteins regulating protein processing, such as sequestosome 1 (SQSTM1/p62) and valosin containing protein (VCP/p97) (34), the latter a previously reported autoantibody target in primary biliary cholangitis (35). It is also possible that the differentiation of the anti-NT5c1A B cell response is reflected in specific epitopes bound by autoantibodies from different diseases. Pluk et al. identified three major autoepitopes by overlapping peptide microarray analyses; the first being near the N terminus (aa 25–50), the second near the C-terminus (aa 341–368) and the third more centrally located (aa 221–243) (4). These findings were confirmed by Larman et al. who identified up to three major immunodominant autoepitopes in similar regions using quantitative dot blot assays: the first near the N-terminus with reactivity against two overlapping peptides aa 30–65 and aa 59–94, the second at aa 204–239, and the third at the C-terminus at aa 334–368 (6). Synthetic peptides representing these autoepitopes were used by others in an ELISA to detect antibodies to NT5c1A (8, 11). Of interest, Herbert et al. noted antibodies to the same epitopes in 20% of SLE and 36% of SjS sera (8), further supporting a common B-cell response. Of note, some sIBM sera found to be positive by immunoprecipitation of the native NT5c1A did not bind the epitopes in a peptide-based ELISA, suggesting that additional immunodominant conformational and/or discontinuous epitopes have yet to be found. Taken together, these findings suggest that the full-length protein as used in the ALBIA of our study, wherein conformational epitopes may be preserved, is a more reliable method to detect anti-NT5c1A in human sera.

Our study also showed a trend whereby sIBM patients with anti-NT5c1A positivity are more likely to have higher disease severity (e.g., muscle weakness) compared to those without anti-NT5c1A antibodies, suggesting these antibodies may have a pathogenic role. However, the pathogenic relevance of anti-NT5c1A is also unclear with respect to phenotypic, pathological and serological differences between seropositive and seronegative groups. A study by Ray et al. found antibodies to desmin but not NT5c1A in plasma cells isolated from sIBM lesions (36). In a passive transfer model, mice injected with anti-NT5c1A antibodies demonstrated macrophage infiltration and significant sarcoplasmic aggregates in myofibers (10). Lloyd et al. (9) reported a lower prevalence of rimmed vacuoles on muscle biopsies of anti-NT5c1A positive patients, but otherwise no apparent phenotypic associations with other clinical or pathological parameters. Tawara et al. suggested a lack of phenotypic differences between groups but a lower incidence of hepatitis C antibodies and a smaller mean area of type 2 myofibers in the seropositive group (10). Another study suggested significant clinical differences between the two groups, citing higher morbidity in anti-NT5c1A positive patients with respect to motor and functional disability, bulbar, facial and respiratory symptoms (7). In a more recent multicenter study of 311 sIBM patients, it was shown that anti-NT5c1A positive patients had a higher adjusted mortality risk (Hazard Ratio 1.89, 95% CI 1.11, 3.21, *p* = 0.019), lower frequency of proximal upper limb weakness at disease onset (8 vs. 23%, adjusted OR 0.29, 95% CI 0.12, 0.68, *p* = 0.005), and an increased prevalence of cytochrome *c* oxidase deficient fibers on muscle biopsy analysis (87 vs. 72%, adjusted OR 2.80, 95% CI 1.17, 6.66, *p* = 0.020) (11). Whether a significant difference in treatment response exists between seropositive anti-NT5c1a and seronegative patients is still inconclusive (10).

We noted that 3/43 (7.0%) of the sIBM patients were positive for anti-SMN antibodies. In these sera, anti-SMN did not overlap with anti-NT5c1A. Autoantibodies directed to SMN were first described in patients with polymyositis/SSc overlap syndrome (37) and we recently reported high levels of anti-SMN in a patient with a severe necrotizing autoimmune myopathy (28). The relevance of this autoantibody in sIBM is unclear at this point, however, since anti-SMN was found in sera that did not have anti-NT5c1A it may help fill a serological gap in sIBM and might provide further insight into an overlap between sIBM and other IIMs.

Limitations of our study include the relatively small size of the sIBM and other disease cohorts, an issue that should be addressed through international multi-center studies using the full-length NT5c1A protein in an ALBIA or equivalent immunoassay as we described here. In addition, these is no established “gold standard” assay for anti-NT5c1A antibodies. We have used immunoprecipitation to validate selected ALBIA positive sera but have found that anti-NT5c1A antibodies are not reliably detected by the radio-labeled IP assay. Nevertheless, a systematic study comparing several immunoassays, including IP are required. Given that antibody isotypes can have differing pathogenic properties, the isotype(s) of the anti-NT5c1A autoantibodies seen in controls and various conditions should be studied in the future. In addition, further studies are required to determine if there is a clinical, pathological, genetic or environmental exposure link that is a common feature of anti-NT5c1A across all conditions where this autoantibody is found. Last, although the majority of sIBM sera were monospecific for anti-NT5c1A in the context of the other autoantibody analyses performed, it is possible that other autoantibodies not included in our study may have confounded the IIF patterns seen.

In summary, we report that anti-NT5c1A autoantibodies as detected by ALBIA have a sensitivity of 48.8% and a specificity of 91.8% for sIBM. In addition, IIF on a commercial HEp-2 substrate has no utility in screening for anti-NT5c1A antibodies, and there may be a relationship between anti-NT5c1A and higher disease severity. Clinicians should interpret positive anti-NT5c1A results in the context of clinical and pathological findings, and exercise caution when applying this biomarker in patients with IIM and other related conditions, such as SLE. Further research is needed to delineate pathogenic mechanisms (if any) of anti-NT5c1A in sIBM and to investigate correlations between high titer anti-NT5c1A seropositivity and disease characteristics.

## Author Contributions

AA designed the study and prepared the written manuscript. MC edited the manuscript and performed statistical analyses. MT and LB edited the manuscript and provided the sIBM sera and relevant clinical and demographic data. AC, CB, and MJ provided SLE sera and edited the manuscript. IG provided IIM sera and edited the manuscript. MM edited the manuscript and performed statistical analysis. HS provided JDM sera and edited the manuscript. MF conceived of the study, provided laboratory work, and edited the manuscript.

### Conflict of Interest Statement

MF is or has been a consultant to and/or has received honoraria or research gifts in kind from Inova Diagnostics (San Diego, CA), Euroimmun Gmbh (Luebeck, Germany) and BioRad (Hercules, CA). AC is a consultant to Exagen Diagnostics (Vista, CA). MM is employed by Inova Diagnostics, a manufacturer of diagnostic autoantibody kits. The remaining authors declare that the research was conducted in the absence of any commercial or financial relationships that could be construed as a potential conflict of interest.
