# Supplementary material for: Anti-NT5c1A Autoantibodies as Biomarkers in Inclusion Body Myositis
Source: Front Immunol. 2019 Apr 9;10:745. doi: 10.3389/fimmu.2019.00745 (PMC6465553; doi:10.3389/fimmu.2019.00745)
Supplement: Supplementary file 1 [file Data_Sheet_1.pdf]

Supplemental Figure 1: Association between anti-NT5c1A antibody levels and muscle weakness. A.) Spearman correlation plot shows significant correlation between anti-NT5c1A antibody levels and muscle weakness (p=0.0178). B.) Patients positive for anti-NT5c1A antibodies had more severe muscle weakness scored on a scale of 1-3 Newton metres (p=0.0356). *Anti-NT5C1A titers divided by 600 (MFU cutoff).

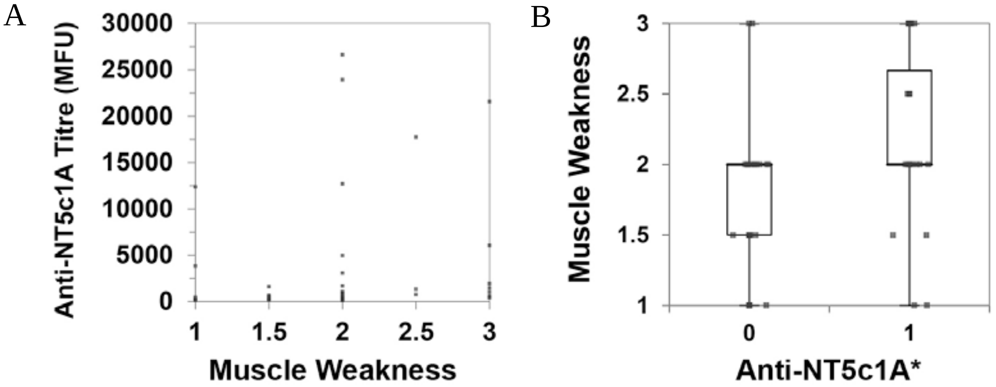

Supplemental Table 1. Data and calculations derived from six eligible publications used for Figure 1 forest plot*.

Authors (Year) [Reference]	Immunoassay	IBM (#) (n)	Pooled Controls (#) (n)	OR	OR-CI	OR+CI	Lower	Upper	Order
Amlani et al (this study 2019)	ALBIA with full length human recombinant protein	48.8% (21) (43)	8.3% (51) (615)	10.556 15	5.43919	20.4869	5.11696	9.93075	7
Salajegheh et al (2011) [5]	IB muscle lysate	52% (13) (25)	0% (0) (40)	87.75	4.84214	1590.22	82.90786	1502.47	6
Pluk et al (2013) [4]	IP of in vitro TnT protein	33% (31) (94)	2.9% (5) (172)	16.434 92	6.11892	44.1428	10.316	27.70788	5
Herbert et al (2016) [9]	ELISA 3 peptides representing major epitopes	37% (88) (238)	3.5% (16) (458)	16.206 67	9.22086	28.485	6.98581	12.27833	4
Lloyd et al (2016) [10]	WB of lysates from transfected HEK cells	60.6% (71) (117)	13.3% (51) (383)	10.047 74	6.2559	16.1379	3.79184	6.09016	3
Tawara et al (2017) [10]	CBA transfected COS cells	35.8% (24) (67)	8.3% (13) (157)	6.1824 69	2.90285	13.1674	3.279619	6.984931	2
Muro et al (2017) [13]	ELISA with IP	80% (8) (10)	7.6% (27) (356)	48.740 74	9.8564	241.027	38.88434	192.28626	1

* See Table 1 for more details
Abbreviations: ALBIA, addressable laser bead immunoassay; CBA, cell based assay; CI, confidence interval; COS, CV-1 in Origin with SV40 genetic material; ELISA, enzyme linked immunosorbent assay; HEK, human embryonic kidney; IB, immunoblot; IBM, inclusion body myositis; IP, immunoprecipitation; JIA, juvenile idiopathic arthritis; n, number of subjects; OR, odds ratio; TnT, transcription and translation of *in vitro* synthesized protein; WB, western immunoblot; #+ve, number positive in anti-NT5c1A assay.

Supplemental Table 2: Summary of ANA IIF on HEp-2 cell substrate

Disease	N	Anti-NT5c1A Positive (%)	ANA Patterns of Anti-NTc51A Positive Sera (*ICAP nomenclature)	Other Myositis Related Antibodies
sIBM	43	21 (48.8)	**Nuclear: 15/21 (71.4%)**<br>67% speckled (AC-2,4,5)<br>33% homogenous (AC-1)<br>5% CENP F-like (AC-14)<br>**Cytoplasmic: 10/21 (47.6%)**<br>5% speckled (AC-19,20)<br>20% Discrete Dots (AC-18)<br>**Mitotic: 6/21 (28.6%)**<br>10% Chromosomal Envelope (AC-28)<br>5% Centrosome (AC-24)<br>5% Intercellular Bridge (AC-27)<br>**Negative: 2/21 (9.5%)** (AC-0)	5% anti-HMGCR<br>7% anti-SMN
IIM	3	1 (33.3)	**Nuclear**<br>100% homogenous (AC-1)<br>**Cytoplasmic**<br>100% AMA (AC-21)	33% anti-PL-7<br>33% anti-NXP2
Statin-related myopathy	4	1 (25.0)	**Negative**: 100% (AC-0)	50% anti-HMGCR<br>25% anti-Mi-2
NMD	13	2 (15.4)	**Nuclear:**<br>100% speckled (AC-2,4,5)<br>**Cytoplasmic:**<br>50% speckled (AC-19,20)	8% anti-SAE<br>8% anti-EJ
Healthy controls	8	2 (25.0)	**Nuclear:**<br>100% speckled (AC-2,4,5)<br>50% homogenous (AC-1)<br>**Cytoplasmic:**<br>100% speckled (AC-18,19,20)<br>50% rods and rings (AC-23)	None

Note: Total % = >100 because some sera displayed more than one IIF staining pattern.
* IIF ANA patterns include CMP cell staining and are referenced according to the standardized nomenclature of the International Consensus on Autoantibody Patterns (ICAP: https://anapatterns.org/index.php).
Abbreviations: AMA, antimitochondrial antibody; CENP-F, centromere protein F; EJ, glycyl-tRNA synthetase; HMGCR, HMG-CoA Reductase; IIF, indirect immunofluorescence IIM, inflammatory immune myopathies; ANA, anti-nuclear antibody; IBM, inclusion body myositis; Mi-2, subunit of nucleosome remodeling-histone deacetylase; NMD, neuromuscular/metabolic disorders; NXP-2, nuclear matrix protein 2; PL-7, threonyl-tRNA synthetase; SAE,

Small Ubiquitin-like Modifier Activating Enzymes; sIBM, sporadic inclusion body myositis; SMN, survival of motor neuron complex
